# The correlation between the mineral drinking water composition and the relevance of dentine in health – A pilot study

**DOI:** 10.12669/pjms.36.3.1820

**Published:** 2020

**Authors:** Creteanu Razvan, Monica Popa, Ana-Maria Incze, Creteanu Cristina

**Affiliations:** 1 Dr. Creteanu Razvan, UMF. Iuliu Hațieganu University of Medicine and Pharmacy, Cluj-Napoca, Romania; 2 Dr. Monica Popa, UMF. Iuliu Hațieganu University of Medicine and Pharmacy, Cluj-Napoca, Romania; 3 Dr. Ana-Maria Incze, RIAI. Research Institute of Analytical Instrumentation, Laboratory of Environmental Analaysis, Cluj-Napoca, Romania; 4 Creteanu Cristina, Pharmacy, Pharmacist at an Independent Pharmacy, Cluj-Napoca, Romania

**Keywords:** Dentine minerals, Minerals in drinking water, Statistical correlations, Public health

## Abstract

**Objective:**

This study estimated the concentration of Ca, Mg and F in drinking water from five counties in Transylvania, Romania and correlated these with mineral values found in the dentine of permanent carious teeth. The role of these minerals on the re-mineralization of teeth is broadly analyzed.

**Methods:**

The study consists of two parts: the first part is a pilot study aimed at determining the concentration of Ca, Mg and F in the dentine of permanent carious teeth of 75 male adults with residence in five counties in Transylvania. The second part is an evaluation of the levels of mineral composition in the drinking water from these counties. Mineral concentrations in dentine and drinking water samples, were determined in the laboratories of the public health institute and the research and analysis institute in Cluj Napoca, Romania.

**Results:**

Statistically we found a direct and significant correlation between the Ca, Mg and F contain from the water samples and Ca, Mg and F contain from the dentine samples and an inverse correlation was statistically highlighted between Ca found in water samples and the F concentration in dentine samples. Improperly water mineralization associated with a general lack of fluoride reveals a potential negative impact on consumer health, including the oral-jaw system.

**Conclusions:**

The results of this study indicate the need for improving the prevention of dental caries which is a priority in promoting orthodontic health for children. The variability in dentine minerals indicates the fact that permanent molar dentin represents an important biomarker for exposure. The future research will have to take into consideration the community residency status and the fact that these studies require a large sample to separate individual and community level contributions to dentin fluoride. Considering these notifications, we conclude that minerals are highly associated with caries.

## INTRODUCTION

Dental caries is chronic, easy to detect and treat in an early but destructive phase after cavitation.^1^ The levels of Ca and Mg in drinking water vary significantly from one source to another. Water treatment processes can affect mineral concentrations and therefore total Ca and Mg intake.^2^

Extensive research has been conducted on fluoride (F) - one of the mineral components in drinking water - and its influence on the mineralized tissue of teeth. Because F has a serious effect on dental caries, the dentists are concerned about its presence in the water. Though Ca and Mg are also present in water, their effect on teeth is limited. It is believed that Ca and Mg are just contributors in enhancing the uptake of F in the oral biofilm. There are also just a few studies on the effects of Ca and Mg on the resistance and susceptibility of teeth to demineralization.^2^,^3^

Dental caries has become a public health issue in the last decades. The problems are rooted in the consumption of fluoridated water and foods prepared with excessive fluoride salt, practices that can cause dental fluorosis. Knowing that there is a linear relationship between the intake of F (mg F/kg/day) and the prevalence of cavities or dental fluorosis, both the concentration of fluoride in drinking water and all other sources of exposure to F must be controlled.^4^,^5^ Our objective was to analyze the role of these minerals on the re-mineralization of teeth.

## METHODS

In a pilot study, a total of 75 permanent teeth of types premolars and molars were collected from 75 adults (males) aged 40-60. The participants were permanent residents of five counties in Transylvania, Romania. All subjects had similar eating habits and similar professional activities. They were healthy subjects who have not been diagnosed with chronic diseases. The subjects were exposed to different levels of F in drinking water.

Permanently carious teeth were examined for cavities by the dentist. They have not practiced local fluoridation treatments or administration of fluoride supplements. The concentrations of minerals found in permanent teeth were determined. Immediately after the dental extraction, the crowns of the teeth were removed, and the dentine was collected.

Under Romanian health legislation, water samples for individual consumption were collected by the beneficiaries from the drinking water distribution network. Ca, Mg and F concentrations in dentine and drinking water samples were determined in the laboratories of the Public Health Institute and the Research Institute for Analytical Instrumentation, Laboratory of Enviromental Annalysis in Cluj-Napoca, Romania. The experimental study lasted several months. To determine the fluoride concentration of the dentine samples, an IC 761 Compact Ion Chromatograph (Metrohm AG, Switzerland), was used. The dentine powder sample was previously treated to remove solids, metal ions and aliphatic organic acids such as mono- or dicarboxylic acids which can interfere with anion separation. The trace mineral of interest was separated by liquid-phase chromatography by applying anion exchange resin as a stationary phase and aqueous solutions of weak mono- and di-basic acids as eluents for isocratic or gradient elution.

Anion exchange chromatography is a way of ion-exchange chromatography, which is used to separate molecules based on their net surface charge. This method uses a positively charged ion exchange resin with an affinity for molecules having net negative surface charges. Anion exchange chromatography is used both for preparative and analytical purposes and can separate a large range of molecules, from amino acids and nucleotides to large proteins.

Detection is carried out with a conductivity detector (CD) because the eluents must have a sufficiently low conductivity. For this reason, the CD is usually combined with a suppressor device (cation exchanger) that will reduce the conductivity of the eluent and will transform the species in the sample into their respective acids. For this purpose, we have used various techniques, including the use of microprobes.

A microprobe represents an instrument that applies a stable and well-focused beam of charged particles (electrons or ions) to a sample. For the determination of the Ca and Mg composition of the dentine of permanent teeth decayed samples, inductively coupled plasma absorption optical emission spectroscopy (ICP-OES) was used.

The Ca and Mg contents in the dentine of permanent teeth decayed samples and the evaluation of mineral composition in water samples were determined using Perkin-Elmer Optima 5300 DV atomic absorption spectrophotometer. The determination of anions and cations in water samples (F-) was performed using a Shimadzu Ion Chromatograph (high-pressure pump isocratic and gradient mode, conductometric detector, UV detector, inside the thermostatic columns, autosampler, suppressor).

The concentration of the two elements were calculated using the calibration curve determined by the instrument for a particular element. These results represent the mean value from 3 measurements.^6^,^7^

### Statistical analysis

All values for quantitative statistical parameters were expressed as mean + standard deviation. A non-parametric correlation analysis (Pearson test and Student test) was performed to determine the relationship between water and teeth composition. A p-value <0.05 was considered statistically significant for correlation analysis and test comparison.^8^

## RESULTS

In the pilot study, Ca, Mg and F concentrations were determined from permanent caries dentine samples taken from N = 75 subjects. The average mineral concentrations obtained are presented in Table-I. We found statistically significant differences between the average concentrations of Ca, Mg, and F in dentine and the Ca, Mg and F concentrations present in drinking water. From samples of drinking water collected from the same residential areas of the participants in the study, we determined the concentrations of Ca, Mg and F (Table-I).

**Table-I:** The average concentrations of Ca, Mg and F (ppm) in dentine samples and Ca, Mg and F in water.

Lot	Dentine Mg average±SD	Dentine Ca average±SD	Dentine F average±SD	Water Mg average±SD	Water Ca average±SD	Water F average±SD
Cj group N=15	3,847.93 ±1,008.35	258,343.34 ±37,458.61	87.73 ±23.45	9.17 ±5.69	27.16 ±16.43	0.13 ±0.07
CT group N=15	4,192.36 ±738.71	303,619.29 ±36,962.69	59.75 ±18.54	11.78 ±3.58	94.82 ±21.27	0.10 ±0.03
SM group N=15	4,526.97 ±694.70	321,861.06 ±31,152.63	79.59 ±15.73	1.29 ±0.58	79.57 ±7.37	0.47 ±0.57
AB group N=15	4,534.60 ± 1,981.61	300,301.38 ±47,435.48	72.33 ±26.67	3.35 ±1.92	14.42 ±4.51	0.18 ±0.17
BM group N=15	4,665.60 ±1,235.28	284,922.35 ±77,970.49	88.54 ±26.86	13.13 ±3.33	31.68 ±12.46	0.41 ± 0.38
Total group N=75	4,353.49 ±1,228.03	293,809.48 ± 52,363.46	77.59 ±24.56	7.74 ± 5.77	49.53 ±34.57	0.26 ± 0.35

The statistical analysis of the average mineral concentration in the analyzed water samples and the mineral content from the samples taken from the local people’s teeth highlighted significant statistical correlation (by using Pearson r correlation) (Table-II).

**Table-II:** Statistical correlations between concentrations of Ca, Mg and F in water samples and dentine samples among investigated groups (Pearson r correlation).

Sample N=75	Water Ca ppm		Water Mg ppm		Water F ppm	

	p	r	p	r	p	r
Dentine Ca ppm	p<0.0006*	0.60*	p<0.0319*	-0.25	p<0.0126*	0.29
Dentine Mg ppm	p<0.87	-0.02	p<0.05	-0.23*	p<0.848	-0.02
Dentine F ppm	p<0.60	-0.22	p<0.5	0.08	p<0.00015*	0.49*

***Note:***

* p < 0.05, r is statistically significant.

**Fig.1:**
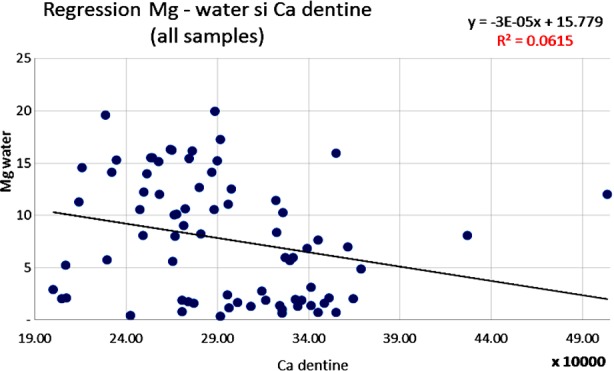
Inverse correlation between Mg level in water samples and Ca level in dentine samples (Pearson correlation).

**Fig.2:**
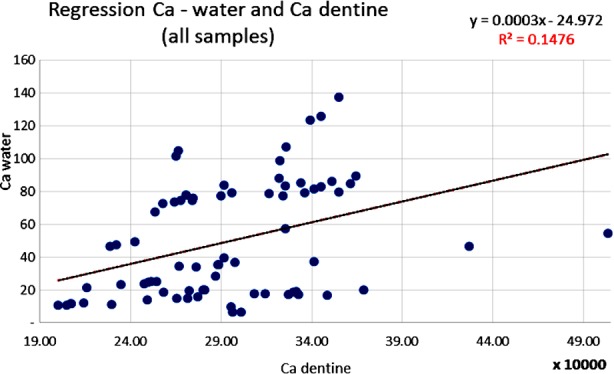
Correlation between Ca level in water samples and the level of Ca in dentine samples (Pearson correlation).

**Fig.3:**
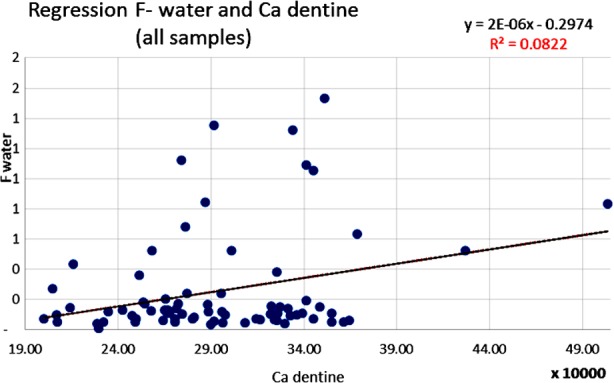
Correlation between F level in water samples and the level of Ca in dentine samples (Pearson correlation).

**Fig.4:**
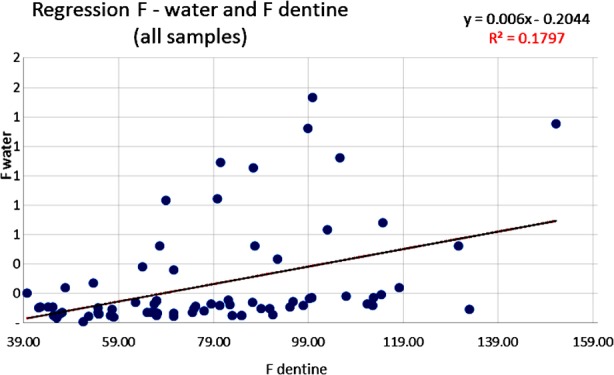
Correlation between F level in water samples and F in dentine samples (Pearson correlation).

The results of this analysis were studied, statistically adjusted and related to dental caries through several linear regression analyses. The statistically significant correlations in Table-II are illustrated by scatter diagrams with regression lines in fig.1,2,3 and 4 for Ca, Mg and F among water samples and teeth samples.

Also, a significant inverse correlation was statistically evaluated when Mg from the drinking water samples was compared to the Ca level in the dentine samples (coefficient r=-0.25; p<0.0319) (Fig.1). A direct correlation has been demonstrated between the Ca level from the water samples and level of the Ca contained in the teeth samples (coefficient r=0.60; p<0.000668). The increase in the Ca concentration in the analyzed water samples reflected an increase of Ca level in the teeth problems which were analyzed (Fig.2).

A statistically significant correlation was highlighted between the level of F contained in the analyzed water samples and the Ca content in dentine samples (coefficient r= 0.29; p<0.0126) (Fig.3). Another statistically significant correlation was highlighted between the level of F contained in the analyzed water samples and the F content in the dentine samples (coefficient r= 0.49; p<0.000151) (Fig.4). A statistically significant inverse correlation was found between the Mg level in water samples and the Mg concentration from the dentine samples analyzed (coefficient r= -0.23; p<0.05).

## DISCUSSIONS

This study shows that the local water in Transylvania has a lower concentration of Ca and Mg than the concentration recommended by the specialists – this is insufficient to promote tooth remineralization, though sufficient levels of F are present to protect against caries.^9^ Fluoride values obtained from the water samples classify Transylvania as fluoride-lacking area. In the analyzed water samples with reduced fluoride concentrations, low concentrations of Ca and Mg were also found, showing reduced overall mineralization of water which together with a general lack of fluoride harms the health of consumers, including oral-jaw system (Table-I).

In all batches, the Pearson coefficient value revealed a high correlation between the Ca and Mg values in the drinking water and the Ca concentration in the subjects’ dentine.Being an essential element for healthy teeth, F is added to drinking water to prevent caries. F concentrations in the studied water samples vary between 0.26±0.35 ppm. Negative and positive types of variations were noticed in the levels of Ca, Mg, and F to the found values in water. Information on metabolic and physiological processes during ontogenetic development is present in enamel. The absence of trace elements in the tissue of the tooth can affect the content of other minerals and may lead to a greater sensitivity of teeth to dental caries.^10^.^11^

Research to determine the concentration of Ca and Mg in dentine is warranted and resolves dentine resistance to noxious agents. The aim of this experiment cannot be achieved in the clinical environment because the method would be too invasive, leading to the destruction of the tooth structure.

The difference of fluoride composition according to the tooth type can be explained by the larger fluoride accumulation time since incisors are the first definite teeth to appear and molars are the last ones, e.g. the wisdom tooth can appear 30 or more years later than the incisors. Higher fluoride concentration in the enamel region of the teeth is associated with increased accumulation of the fluoride in the dentine part of the teeth.

F concentration of dentine reveals a biomarker for cumulative fluoride exposures. F concentration of the dentine of permanent teeth for lifelong residents is correlated with the fluoride concentration of the community water supply and consumption of high-fluoride containing food products.^12^

The level of exposure for an individual via drinking water is directly proportional to the level of water fluoride and the daily amount of water intake. Fluoride has been shown to reduce dose-dependent molar crowns formed in the dentine and to increase the risk of caries. Fluoride absorption is correlated with the tubular structure of dentinal fluid and metabolic activity of dentine. This makes it possible for fluoride ions to have an odontoblastic effect on the formation of dentine. Daily increased consumption of mineral water in addition to the public water supply could have implications for the safety of fluoride supplementation.

Drinking water with the recommended concentration of Ca, Mg, and F creates a positive milieu in the oral cavity for promoting remineralization processes in mineralized tissue. Information about the mineral content of the drinking water and their health significance is essential to both public and health-care professionals.^13^

## CONCLUSIONS

The investigated water samples from Transylvania have a low degree of mineralization, both in terms of macroelements (Ca, Mg) and some microelements, specifically F. The fluoride input, although not increased by the water in the network, must be taken into account for cumulated consumption (network water, mineral water or other F sources).

Transylvania is characterized as an area deficient in minerals and therefore tooth decay rate is increased. Water quality and socio-economic decay influence the population. The results of these analyses were studied, statistically adjusted and related to dental caries through several linear regression analyses.

Multivariate analysis revealed three of the variables independently correlated with the development of carious lesions: concentration of fluoride in the water, poor overall mineralization of water and the increased levels of minerals in dentine for the teeth from the exposed individuals. The strong effect of the geographical location shows that these communities have unique characteristics which are relevant determinants of minerals and fluoride in dentine, regardless of the individual characteristics. These results are important for the dentists in Transylvania for prophylaxis of tooth decay and dental fluorosis.

The variance in dentine minerals seems to indicate that permanent molar dentine may be a promising biomarker for exposure. This study suggests that a major source of variability in dentine minerals is the community itself, independent of water fluoridation status.

Health education is a communication activity, chasing the improvement of health and the prevention or mitigation of diseases, individually or collectively, by influencing conceptions, attitudes, and behavior with administrative factors and involvement of the community.

### Authors’ Contributions:

**CR:** project development, data collection, manuscript writing and responsible for the integrity of the work.

**MP:** data analysis.

**AI:** sample analysis, data analysis.

**CC:** data analysis, statistical correlations, article editing.
